# A novel approach to model traffic on road segments of large-scale urban road networks

**DOI:** 10.1016/j.mex.2019.04.024

**Published:** 2019-04-29

**Authors:** Amila Jayasinghe, Kazushi Sano, C. Chethika Abenayake, P.K.S. Mahanama

**Affiliations:** a Department of Town & Country Planning, University of Moratuwa, Moratuwa, 10400, Sri Lanka; b Urban Transport Engineering and Planning Lab, Nagaoka University of Technology, Nagaoka, 940-2137, Japan

**Keywords:** Space Syntax, Traffic modeling, Centrality, Space syntax, Network analysis, Developing countries

## Abstract

The study proposes a novel method for modeling traffic volumes at the road segment level of large-scale urban road networks. This study has been placed in a milieu where existing methods on modeling vehicular traffic volume are hampered by data and cost constraints, especially in developing countries. Emerging traffic modeling methods, based on centrality and space syntax provides a technically-efficient approach to overcome the above-mentioned constraints. Nevertheless, those methods are yet to be popular among practitioners due to limited accuracy and validity. This study modifies the existing methods and validates in five case cities to make them practice-ready. Findings of this study indicated that the proposed method is competent enough to estimate traffic volume of road segments on a par with the internationally accepted standards.

• The proposed method combines two network centrality measures abstracting the traffic volume on a road segment as the sum of origin-destination trips (i.e., Closeness-Centrality) and pass-by trips (i.e., Betweenness-Centrality).
• The study modifies the ‘distance’ variable in the existing formula as 'path-distance' which captures topological and mobility characteristics of roads.
• The method does not require extensive data and can be implemented by utilizing publicly available open-source network analysis software, hence, ideal for resource-scarce situations.

## Graphical abstract

**Figure:**
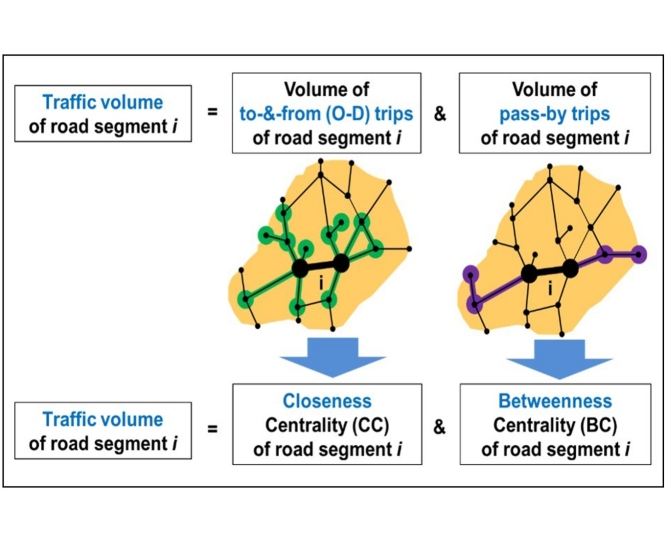

**Specifications Table**

**Table:** 

Subject Area:	*Engineering*
More specific subject area:	*Traffic and Transport Engineering*
Method name:	*Space Syntax*
Name and reference of original method:	*Hillier, B., 1999. The common language of space: A way of looking at the social economic and environmental functioning of cities on a common basis. Journal of Environmental Sciences-Beijing, Volume 11, pp. 344-349.*
Resource availability:	*N/A*

## Method details

The rapid urbanization in developing countries has led to a marked leap in traffic volumes and caused several problems such as traffic congestion, road accidents, and air pollution [1,2]. Solving those problems has become a strong challenge due to high investment costs as well as the limited availability of accurate [[3], [4], [5]] and up-to-date data on traffic volume. This demands a strategic, quick and cost-effective solution to identify the existing traffic volumes and to model the trajectories of future scenarios in developing countries (Hassan & Hoque, 2008), (Walker et al., 2010), (Fujiwara & Zhang, 2013).

A continuous record of the traffic volume data collected throughout the year is the most reliable input for obtaining traffic volume. However, it is not economically feasible to fully install Automatic Traffic Recorders (ATRs) for extensive road networks in developing countries. As an alternative, coverage count method (also called as the traditional factor approach) is widely utilized in estimating traffic [6]. However, it is still not economical enough for all road segments in a macro network (i.e. region, metropolitan area) [6,7]. Further, coverage count is only suitable to estimate existing traffic volume and not able to model future scenarios. Therefore, many researchers have attempted to develop alternative methods to model traffic volume without using extensive traffic count data, and those methods predominately belong to ‘direct demand modeling’ and ‘multi-step travel demand modeling’ [7,8].

Direct demand models (also called as the regression modeling) estimate traffic volume based on a set of explanatory variables including roadway characteristics, land use characteristics and socioeconomic factors. Research works of, Mohamad et al.,’s [9], Zhao and Chung [6], Lowry [10] Doustmohammadi et al.,’s [11] developed series of regression models with a satisfactory estimation of traffic volume. However, applicability of the above-mentioned methods were questioned in the context of developing countries as well as small and medium-size cities due to the unavailability of continuous, short-interval, micro-data for significant predictors such as long-term socio-economic conditions [12,13]. Further, direct demand models have mostly considered the localized characteristics of roadways (i.e. functional category, roadway surface, access locations to highways) and unable to conceptualize road network as a system [14]; and ignore the mutual interactions between land use and transport system.

Methods that have been developed based on the multi-step travel demand modeling approach are considered as the most advanced application in traffic volume estimation and travel demand modeling. The very first version of multi-step travel demand modeling is ‘four-step land use transport model.' Next generation of this modeling approach is ‘integrated urban land use transport models. Conventional four-step models and integrated urban land use transport models are considered as an aggregated trip-based models because, it accounts travel as a function of the size of a zone and travel demand as a function of trips than of activities. Recently, multi-step travel demand modeling has been shifting towards disaggregated trip-based models, tour-based models, and activity-based models. As a result, activity-based models have become a popular application in transport engineering and planning, [15]. Many researchers and policymakers have highlighted that adopting multi-step modeling in the context of developing countries is constrained due to inadequate, up-to-date land-use and O-D trip data; lack of financial resources and inadequate technical expertise [[16], [17], [18], [19],^15^,^20^].

Other methods utilize to estimate traffic volume are; image-based data such as high-resolution satellite images and aerial photographs [21], machine learning algorithms such as Artificial Neural Network (ANN) and location-based social network data such as social media, GPS, Bluetooth data. Nevertheless application of those methods are also limited due to cost constraints on purchasing and processing image data [22,12]; the requirement of an extensive baseline data and more complex statistical procedures that demand high technical competence for the calibration of machine learning algorithms [12,23]; and lack of big data and limited online users that makes the sample size too small for application of location-based social network data [21,24,25].

There is a need for an alternative method to identify the existing traffic situation and the model future scenarios that can efficiently work under the above-mentioned data, cost and technical know-how constraint situations. In catering to the above need, this study focused on a set of research literature related to network centrality measures. Centrality measures, which have been evolved from graph theory, were initially a popular concept, in the fields of social network analysis and computer engineering, that applied to explain matters related to accessibility [26], (Chen et al., 2014), [27,28]. The results of previous works have repeatedly claimed that the centrality is capable to explain pedestrian and vehicular flows [29,30], [31,32], [33,34,7,35].

Even though the results of the previous studies have provided a green light, many challenges are yet to overcome when employing centrality measures to model vehicular traffic volume at the road segment level. Previously conducted space syntax-based researches have revealed a significant correlation with vehicular traffic (R^2^>0.6) and a moderate correlation with pedestrian traffic (R^2^<0.6) [36]. In those studies, the link cost of explanatory variables was primarily referred to the cognitive behavior of human movements (i.e., topological shortest path, the least angular turns) and the influence of the roadway characteristics such as mobility, traffic congestion, and network uniqueness have not been considered much [36]. Previously conducted studies related to vehicular traffic volume and centrality have well considered the flow of pass-by trip distribution yet have not exploded land use generated trips in relation to centrality [[36], [37], [38]]. Lowry’s [29] works on AADT estimation by employing ‘origin-destination centrality’ also could not solely rely on centrality measures as relative ‘trip production/attraction potential’ values were derived from land use data. Utilizing land use data in developing countries is difficult due to the lack of availability of updated land use maps and the resource consuming nature of the data collection. Jiang et al.’s (2008) [32] works found a significant correlation between traffic flow and name streets centrality whereas less correlation with road segment-based centrality. “Previous studies have indicated that topological measures (e.g. centrality) can be used to predict traffic flow at the aggregate level but mainly focus on correlation analysis based on traffic data such as AADT data” [39]. Pun et al. [39], has attempted to estimate traffic volume through integrating topological and geometrical properties using AI-based multiple regression models. Results have revealed that a combination of topological and geometrical measures results in higher R values (R = 0.66) but still lacks the accuracy to model traffic flow. Therefore, there is a need to further look at the applicability of network centrality to model traffic volume on road segments at macro level road networks.

With due consideration, the objective of this study is to develop a network centrality-based method to model the vehicular traffic volume of road segments at macro level road networks. Pun et al., [39] have generated a statistically best-fit multiple regression model by combining five centrality measures. The integrated centrality measure proves better predictability (R^2^ = 0.6) compared to sole centrality measures. However, this does not provide any theoretical underpinnings on the properties of each measure and its reflections over the integration. The proposed method attempts to overcome the above-mentioned challenges by incorporating two novel features. The approach of this study was to theoretically explain the traffic flow by integrating two centrality measures at first and then to statistically validate that based on the empirical data. Traffic volume at a given location of a road is the total number of vehicles (or persons) that move through the given location at a specific time. In other words, the traffic volume of a given location is the sum of vehicles pass-by, starting a trip and ending a trip at the given location. The method proposed to compute the traffic volume has utilized betweenness centrality (BC) and closeness centrality (CC) to capture ‘pass-by’ and ‘O-D’ trips respectively. Secondly, the study introduced ‘path-distance’ (PD) variable to capture the mobility characteristics and the roadway characteristics of the road network when computing the distance. The method has been validated in five different case cities from developing countries. Unlike existing traffic volume estimation and travel demand modeling methods, the proposed method requires neither huge database (i.e. land uses, O-D trip and extensive AADT counts) nor an expensive software, hence, ideal for the resource-scarce situations, particularly, in developing countries.

The paper is organized into four sections. The introduction section summarizes the literature survey on the applicability of existing traffic volume estimation and travel demand modeling methods in the context of developing countries. The second section provides a description of the method. That section includes a brief description of five case studies and the concepts of the proposed method. Results of the study explain the relationship between traffic volume and centrality measures computed by the proposed method. Thirdly, the study validates the proposed method’s capability to estimate traffic volume. The fourth section notes the conclusion with recommendations on applying the proposed method.

## The method

The study proposes a network centrality-based method to model the vehicular traffic volume of road segments at macro level road networks. The study suggests that the traffic volume of a road segment be equal to the sum of the volume of the trips originated from and ended at a road segment (i.e., O-D trips); and the pass-by trips within the given road segment (refer Eq. (1)). Accordingly, the study utilizes closeness centrality (CC) to capture the volume of O-D trips of a road segment (refer Eq. (2)) and betweenness centrality (BC) to capture the volume of pass-by trips of the given road segment (refer Eq. (3)).

(1) Traffic volume of road segment (i) = Volume of O-D trips of road segment (i) + Volume of pass-by trips of road segment (i)

(2) Volume of 'O-D trips' of road segment (i)= f[CC(i)]

(3) Volume of 'pass-by trips' of road segment (i)= f[BC(i)]

Closeness centrality explains, “the notion of accessibility of a location [road segment] and measures how close the location [road segment] to all others along the shortest path” [40]. Previously conducted space syntax-based researches have already revealed a direct relationship between accessibility [closeness] and the volume of land use generated trips [O-D trips] [29,41]. BC captures “a special property in a particular location [road segment] that does not act as either origin or destination but as a pass-by location” [40]. The CC of a node is the inverse of the average distance from this node to all other nodes whereas BC of a node is the number of shortest paths between two nodes that contain the given node [40]. Classically, the centrality of road networks is modeled with two methods as primal graph and dual graph, wherein primal graph nodes illustrate junctions, and in the dual graph, nodes illustrate roads as a means of giving importance to roads’ segments [42]. As the focus of this method is road segments, not the junctions, the dual graph method was employed. In duel graph method, CC of a road segment as the inverse of the total distance from this segment to all other road segments in the entire planar graph network; and BC of a road segment as the sum of geodesics that pass through a given road segment in the entire planar graph network [32,43,39]. Centrality measures are computed as normalized values as well as non-normalized values depend on the purpose of the study. In previous studies, two methods were adopted to normalize; based on the total number of links/nodes in the network (Refer Eqs. (4a) & (5a)) [40] and the radius of the considered node to the network influence area (Refer Eqs. (4b) & (5b)) [44]. Normalized equations are utilized for comparing nodes or road segments with the other networks volume-based ranking [45]; and for temporal analysis of the same network such as volume change forecasts ([46,47];) see Ahmadzai, Rao and Ulfat (2018) for further details with regards to the normalized centrality. The study utilized Chiaradia et al’s (2013) [43] formula to compute CC and BC of road segments (refer Eqs. (4) and (5) respectively).

(4) $$\text{C}\text{C}\;(\text{i})=\;\frac{1}{\sum_{\;\text{j}\neq \text{i}}{\text{d}}_{ij}}\;$$

(4a) $${\text{C}\text{C}}_{\text{n}\text{o}\text{r}\text{m}}(\text{i})=\;\frac{\left(N-1\right)}{\sum_{\;\text{j}\neq \text{i}}{\text{d}}_{ij}}\;$$

(4b) $${CC}_{norm}^{r}(\text{i})=\;\frac{1}{\sum_{\;\text{j}\neq \text{i}}{\text{d}}_{ij}}\;$$

(5) $$\text{B}\text{C}\;(\text{i})=\sum_{\text{j}\neq \text{i}\neq \text{k}}{\text{p}}_{jk}\left(i\right)/{\text{p}}_{jk}\;$$

(5a) $${\text{B}\text{C}}_{\text{n}\text{o}\text{r}\text{m}}(\text{i})=\;\frac{2}{\left(N-1)(N-2\right)}\sum_{\text{j}\neq \text{i}\neq \text{k}}{\text{p}}_{jk}\left(i\right)/{\text{p}}_{jk}\;$$

(5b) $${BC}_{norm}^{r}(\text{i})=\;\sum_{\text{j}\neq \text{i}\neq \text{k}}{\text{p}}_{jk}\left(i\right)/{\text{p}}_{jk}$$

(6) $$\text{T}\text{V}\;(\text{i})\;\;=\;\text{a}\;+\text{b}\text{*}[\text{C}\text{C}\;(\text{i})]+\;\text{c}\text{*}[\text{B}\text{C}\;\left(\text{i}\right)]$$

Where,

CC (i) = Closeness centrality of road segment ‘*i’,*

${\text{C}\text{C}}_{\text{n}\text{o}\text{r}\text{m}}(\text{i})$ = Normalized closeness centrality of road segment ‘*i’ for undirected graph,*

${CC}_{norm}^{r}(\text{i})$ = Normalized closeness centrality of road segment ‘*i’ within the search radius r,*

BC (i) = Betweenness centrality of road segment ‘*i’,*

${\text{B}\text{C}}_{\text{n}\text{o}\text{r}\text{m}}(\text{i})$ = Normalized betweenness centrality of road segment ‘*i’ for undirected graph,*

${BC}_{norm}^{r}(\text{i})$ = Normalized betweenness centrality of road segment ‘*i’ within the search radius r,*

N = Total number of road segments [i.e. nodes in dual graph] in the network,

${\text{d}}_{ij}$ = Distance between road segments ‘*i’* and ‘*j’* along the shortest path,

${\text{p}}_{jk}(i)$ = Number of geodesics [shortest paths] between road segments ‘*j’* and ‘*k’* that passing through road segment ‘*i’,*

${\text{p}}_{jk}$ = Number of geodesics [shortest paths] between segments *‘j’* and *‘k’*,

TV (i)= Traffic volume of road segment ‘*i’,*

a, b & c are constants.

Accordingly, the traffic volume of road segment *i* can be expressed as follows (refer Eq. (6)). In the proposed model, betweenness and closeness centrality are the explanatory variables of the traffic volume that simulates O-D trips and pass-by trips respectively. Thus, it replaces all four stages of the traditional transport model. Theoretically, BC performs a key role in explaining traffic volumes in locations where there are more pass-by trips such as regional roads that connect residential townships to a major city. Nevertheless, the model is incomplete without CC because CC is the key to explain the variations in traffic volumes at trip-generation locations such as shopping districts.

As indicated in Eqs. (4) and (5), the type of distance utilized to identify the ‘shortest path’ and the unit of distance between road segments are crucial factors in computing BC and CC. Some of previous studies utilized unit distance and angular changes as the distance when computing centrality [34,[48], [49], [50]]. In contrast, Paul [36] and Lowry [7] have highlighted the importance of an impedance factor to account not only topological distance but also mobility characteristics of roadway units and roadway characteristics such as network uniqueness, land use access opportunities, and traffic congestion. Hillier, [29], Hillier and Iida (2005) [31], Dabaghian et al. [51] and Javadi et al. [52] have argued that the behavioral implications of the travelers’ knowledge on road network are more related to visual and topological properties of the network than mere travel time. Further, in the fields of traffic and transport planning and engineering, link cost in route choice modeling is often expressed by travel time [53,54]. Primarily, three kinds of methods are employed in estimating travel time for route choice modeling, i.e., free flow travel time, congested travel time and time-dependent travel time. However, studies on route choice behavioral mechanisms have suggested incorporating the traveler’s perception of travel time instead of purely depending on real travel time (Sumalee et al., 2009), (Parthasarathi et al., 2013), [55,56].

While taking into account the above-mentioned research findings and arguments, when computing the distance factor in CC and BC, this study utilizes the angular change-adjusted metric distance (MD). Most of the previous studies did not take the geometric weights of node and segment or both into consideration, nevertheless, in recent studies, it has been popular as an attempt for further accuracy [42]. Though there are no segment-based weight (such as road class, road speed, road volume, and road width) adopted in relation to traffic volume studies so far [42], it has been proved to produce better results in previous works on the impact of street centrality over land use [44]. This method proposes a segment-based weight that can take travel time into account in calculating the distance. Hence, MD between two road segments was proposed to be normalized by the average speed. As speed data is difficult to be obtained at the road segment level, the average speed of road segment was assigned based on the road type (Ty). Accordingly, this study proposes ‘path distance’ (i.e. PD), which is a function of the average speed by road type (Ty) and the angular change-adjusted metric distance (MD) (refer Eq. (7)) in computing BC and CC. MD measures the length of the road segment in kilometer. Table 1 introduces the assigned Ty values by road types along with the average speed. The combined effect of MD and Ty can account mobility characteristics as well as roadway characteristics (i.e. Expressways, Arteries, Sub- Arteries, Collectors, and Local roads).

(7) $${PD}_{\;}=Ty*MD$$

**Table 1:** Suggested Ty values by road hierarchy.

Road type	Characteristics	Average Speed (km/h)	Ty
Expressways	Toll roads and controlled-access highways. Number of lanes is less than 6 with a central median	70–100	1/80
Major arteries	Connects two or more provincial/state capital cities. Number of lanes is 4 to 6 with a central median	50–70	1/60
Minor arteries	Connects the medium and small towns within a province/state. The number of lanes are 2 to 4 without a central median.	30–50	1/40
Collectors	Connects local areas to a medium or small town. The Number of lanes is 2 without a central median.	20–30	1/25
Local roads	Connects neighborhood residential areas to collectors. Single lane without a central median.	<20	1/15

The study utilized the Spatial Design Network Analysis (sDNA) tool [43] in Geography Information System (GIS) environment to compute BC and CC. The tool requires a ‘network graph’ file as the input and ‘analysis option’ to compute BC and CC. This study utilizes ‘road-segments’ graph method [30] to convert the real road network into the network graph. In road-segments graph method, the road segments are termed as ‘links’ and the road intersections are termed as ‘nodes’. For preparing the graph, the study utilized ‘road centerlines,' i.e., vector line data that represent the geographic center of the rights-of-way on the given road segment. In the graph, segments represent physical locations of trip origins and destinations.

The ‘analysis option’ is the method of calculating the shortest-path. The study utilized the ‘custom distance’ as the analysis option and entered PD as the custom distance input. Accordingly, the study prepared an attribute to represent the custom distance by multiplying the metric distance of road segment by the assigned utility value as per the road type (i.e., Eq-7; PD = MD * Ty). The considered radius of a node to the network influence area is 20 km (i.e. r = 20 km). Accordingly, the traffic volumes at the road segment level were estimated by the proposed model (Eq. (6)). In order to validate the model, empirical data on AADT values (i.e., real-world traffic volumes) of over 7000 road segments were obtained from the JICA database. The comparison of empirical traffic volume data and modeled traffic volume data are discussed in the validation sections.

## Validation

The proposed method was tested in five case study areas, namely Colombo, Phnom Penh, Hanoi, Karachi and Dares Salaam (Table 2). All case study areas are fast growing capital cities which are located in five developing countries in Asia and Africa. Five case study areas manifest unique road patterns, which enables to investigate the applicability of the proposed method in any given geographical area. Table 2 gives a brief description of the case study areas.

**Table 2:** Case study areas.

Case Study Area	Colombo	Phnom Penh	Hanoi	Karachi	Dares Salaam
Country	Sri Lanka	Cambodia	Vietnam	Pakistan	Tanzania
Area (sqkm)	995.54	678	921	3,700	1,590
Population	5.8 million	1.7 million	3.2 million	18 million	4.3 million
Network length (km)	2,075	820	950	1,150	785
Trip rate per person	1.87	2.51	2.66	1.36	–
GDP (2017)	$48 billion	$24 billion	$40 billion	$113 billion	–
Year	2013	2012	2007	2010	2008
Road pattern	Radial	Grid-Radial-Ring	Grid-Radial	Radial-Ring	Radial-Ring

These case study areas were chosen by considering the available data on traffic volume. Traffic volume is the response variable in the proposed model. The study obtained traffic volume data from secondary sources. Traffic volume has been reported as Annual Average Daily Traffic (AADT), converted to Passenger Car Unit (PCU) per day using the recommended AASHTO (American Association of State Highway and Transportation Official) PCU factors. AADT values and road network for Colombo were collected from ‘CoMTrans Urban Transport Master Plan-JICA database’ and for other four areas from ‘Person Trip Survey – JICA database.' The above-mentioned databases consist of traffic volume data collected by sample surveys over 7000 road segments within 5 case study areas (i.e., 1927 road segments in Colombo, 1279 road segments in Phnom Penh, 2397 road segments in Hanoi, 1128 road segments in Karachi and 679 road segments in Dares Salaam). For model formulation purpose, the study employed regression analysis and utilized Ordinary Least Squares Regression (OLS), Robust Regression (RR) and Poisson Regression (PR) statistical techniques. After checking the multicollinearity among explanatory variables, the study utilized R^2^ and Median Absolute Percent Error (i.e., MdAPE) to test the goodness-of-fit when selecting the most suitable model. R^2^ and MdAPE together provide a sound understanding of the predictability of the model [7]. The study has initially utilized randomly selected 80% of the data for calibration (i.e., a random subset of calibration data) and 20% to validation.

The regression analysis indicated that the proposed model comprised of BC and CC as explanatory variables produces a higher goodness of fit values (R^2^ > 0.9) and lesser Percent Root Mean Squared Error (RMSE < 20%) compared to the model comprised of BC as the explanatory variable (R^2^ < 0.8, RMSE > 40%). As per the international AADT modeling standards, (i.e., [57]) R^2^ value should be greater than 0.85 and the RMSE should be less than 30%. Hence, the model comprised with BC and CC both as explanatory variables were considered as the best regardless the strong similarity of the BC and AADT as well as the lower weight in the regression coefficient the intercept obtained by CC (b*BC >> c*CC). Table 3 illustrates the statistics and specifications of the best model to estimate AADT for each case study area. The recorded R^2^ values of each case study area were more than 0.90 for calibration and validation, and there was no significant multicollinearity among explanatory variables. Further, MdAPE values of the models were less than 20% for calibration and validation respectively.

**Table 3:** Statistics and specifications of the best model for each case study areas.

Specifications		Case study area				
		Colombo	Phnom Penh	Hanoi	Karachi	Dares Salaam
Coefficient value						
a (Constant)		3.437 (p < .0001)	3.906 (p < .0001)	3.420 (p < .0001)	3.924 (p < .0001)	3.212 (p < .0001)
b (for BC_PD_)		0.594 (p < .0001)	0.561 (p < .0001)	0.543 (p < .0001)	0.574 (p < .0001)	0.678 (p < .0001)
c (for CC_PD_)		2.002 (p < .0001)	1.853 (p < .0001)	1.532 (p < .0001)	1.761 (p < .0001)	1.574 (p < .0001)
Presence of multicollinearity						
Tolerance		0.770	0.940	0.235	0.570	0.987
VIF		1.299	1.063	4.253	1.754	1.013
Goodness-of-fit						
Calibration*	N	1542	1009	1918	902	543
	R^2^	0.928	0.936	0.916	0.977	0.967
	Adjusted R^2^	0.908	0.931	0.915	0.944	0.953
	MdAPE	17.99%	16.58%	13.8%	12.6%	18.4%
Validation**	N	385	270	479	226	136
	R^2^	0.935	0.942	0.923	0.951	0.959
	MdAPE	17.15%	15.94%	12.8%	10.5%	17.3%
Correlations – AADT and Explanatory Variables in the Model						
Partial	BC	0.77	0.78	0.76	0.79	0.78
	CC	0.57	0.58	0.57	0.59	0.59
Part	BC	0.7	0.71	0.71	0.71	0.71
	CC	0.51	0.52	0.51	0.52	0.52
(Partial^2)%	BC	59%	61%	58%	62%	61%
	CC	32%	34%	32%	35%	35%
(Part^2)%	BC	49%	50%	50%	50%	50%
	CC	26%	27%	26%	27%	27%

*Note*: Response variable AADT, a, b and c are constant values (refer Eq. (6)).

* Random 80% of the sample.

** Random 20% of the sample.

This R^2^ and MdAPE values are on a par with the results of previous works on modeling AADT [6,7,23], by other standard methods. Further, the study computed the RMSE for each case study area and compared with the international standards (i.e. [57]) (refer Table 4). Results indicated that the recorded RMSE values by AADT categories for all five case study areas were in line with the international standards. Hence, these results are versatile enough to recommend the developed models based on network centrality in estimating AADT in any AADT category.

**Table 4:** Recorded RMSE by AADT categories for each case study areas.

AADT categories	RMSE as per FHWA*	Colombo		Phnom Penh		Hanoi		Karachi		Dares Salaam	
		RMSE	N	RMSE	N	RMSE	N	RMSE	N	RMSE	N
> 50,000	10	9.3%	105	7.1%	138	9.6%	251	9.4%	290	7.4%	57
25,000 – 50,000	15	13.0%	241	16.9%	338	13.5%	515	15.0%	273	13.8%	39
10,000 – 25,000	20	24.9%	538	19.0%	223	20.1%	1368	21.0%	403	19.4%	105
5,000 – 10,000	25	16.4%	311	27.7%	438	25.8%	258	24.8%	162	22.4%	177
2,500 – 5,000	50	22.6%	202	39.0%	115	48.3%	5	–	–	37.3%	297
1,000 – 2,500	100	27.7%	274	–		–	–	–	–	78.5%	4
< 1,000	200	193.1%	256	412.5%	27	–	–	–	–		
Average	30	19.1%	1927	18.0%	1279	16.1%	2397	14.2%	1128	18.4%	679

**Fig. 1:**
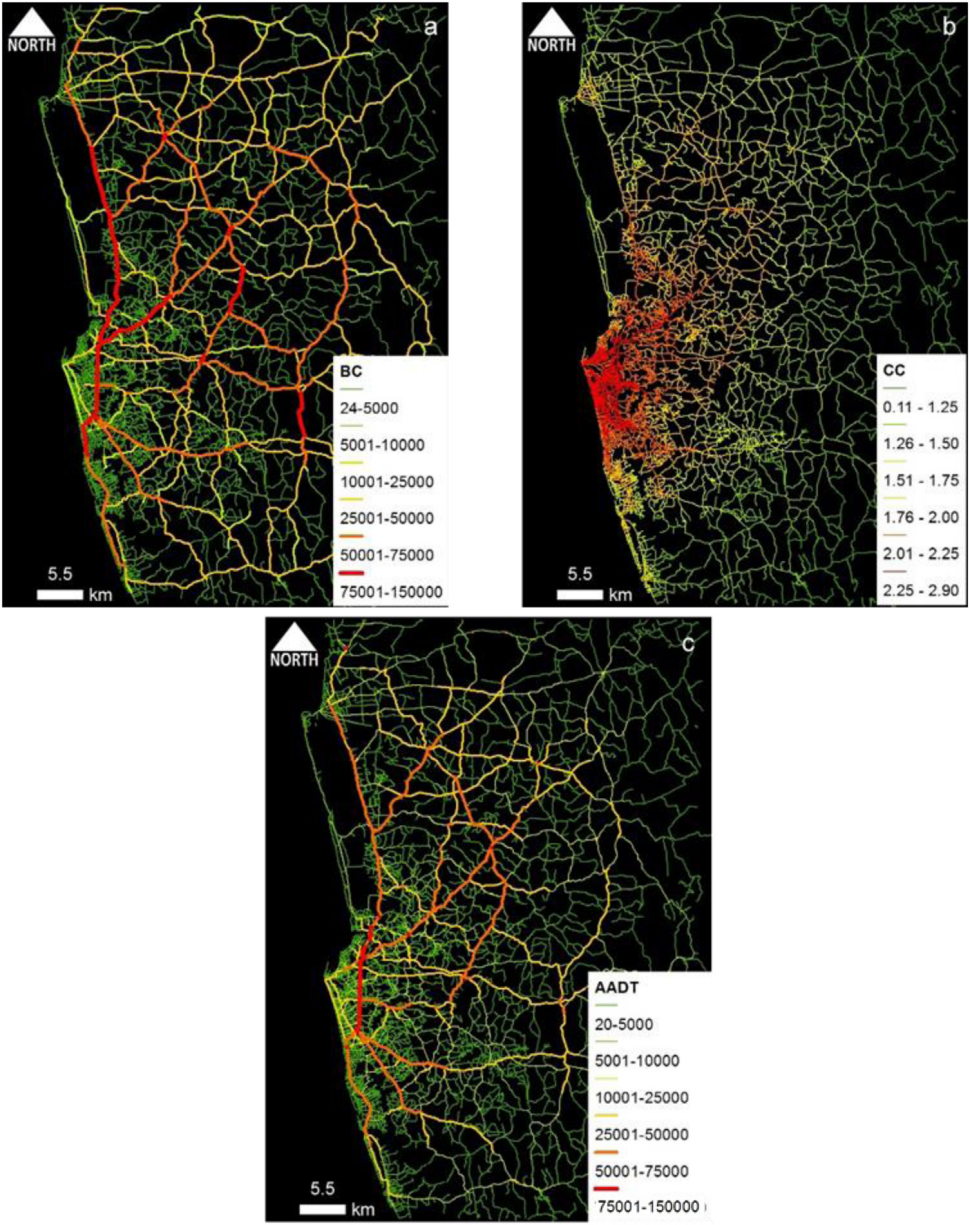
Colombo case study area: Spatial distribution of (a) BC_(PD)_ and (b) CC_(PD)_ and (c) estimated AADT.

**Fig. 2:**
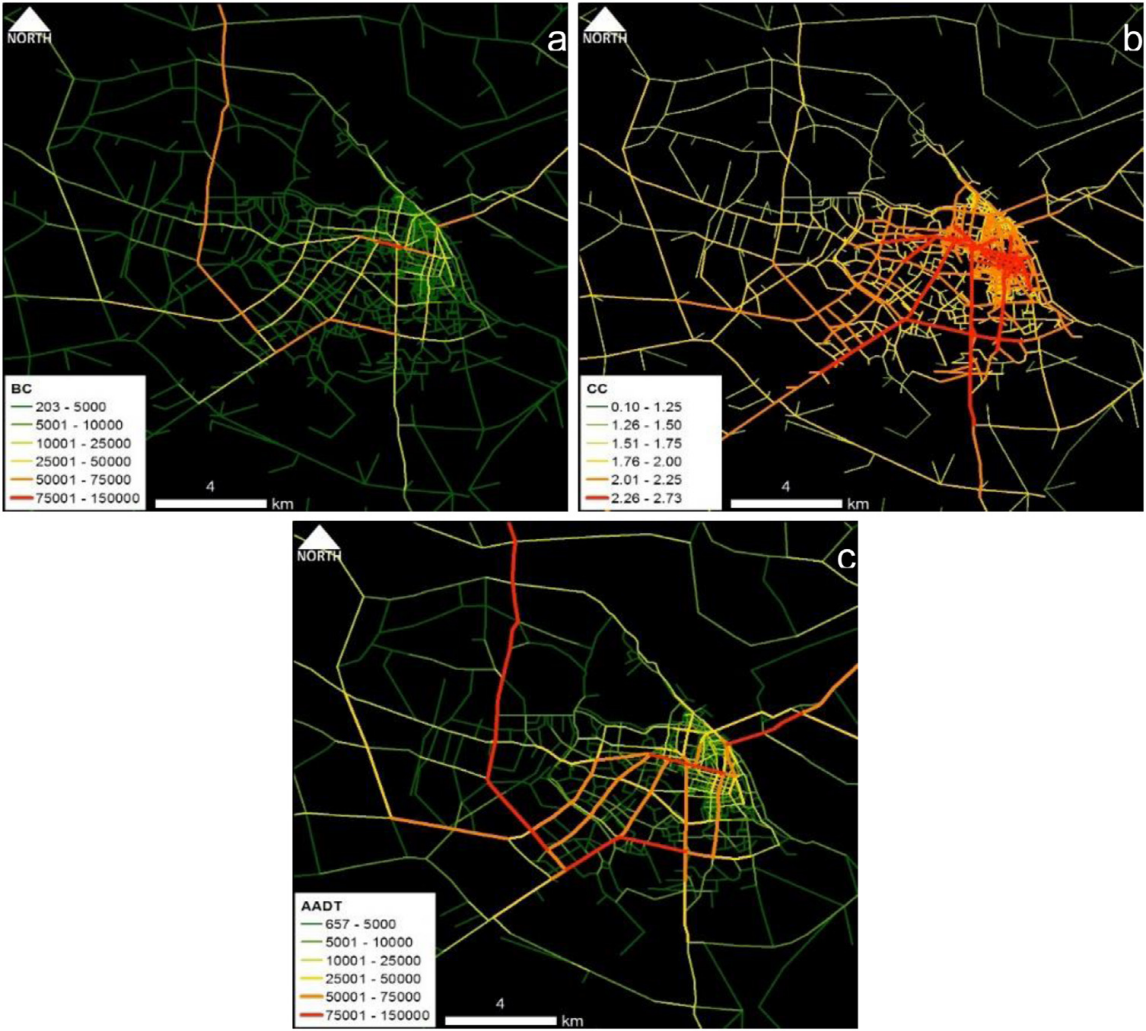
Phnom Penh case study area: Spatial distribution of (a) BC_(PD)_ and (b) CC_(PD)_ and (c) estimated AADT.

**Fig. 3:**
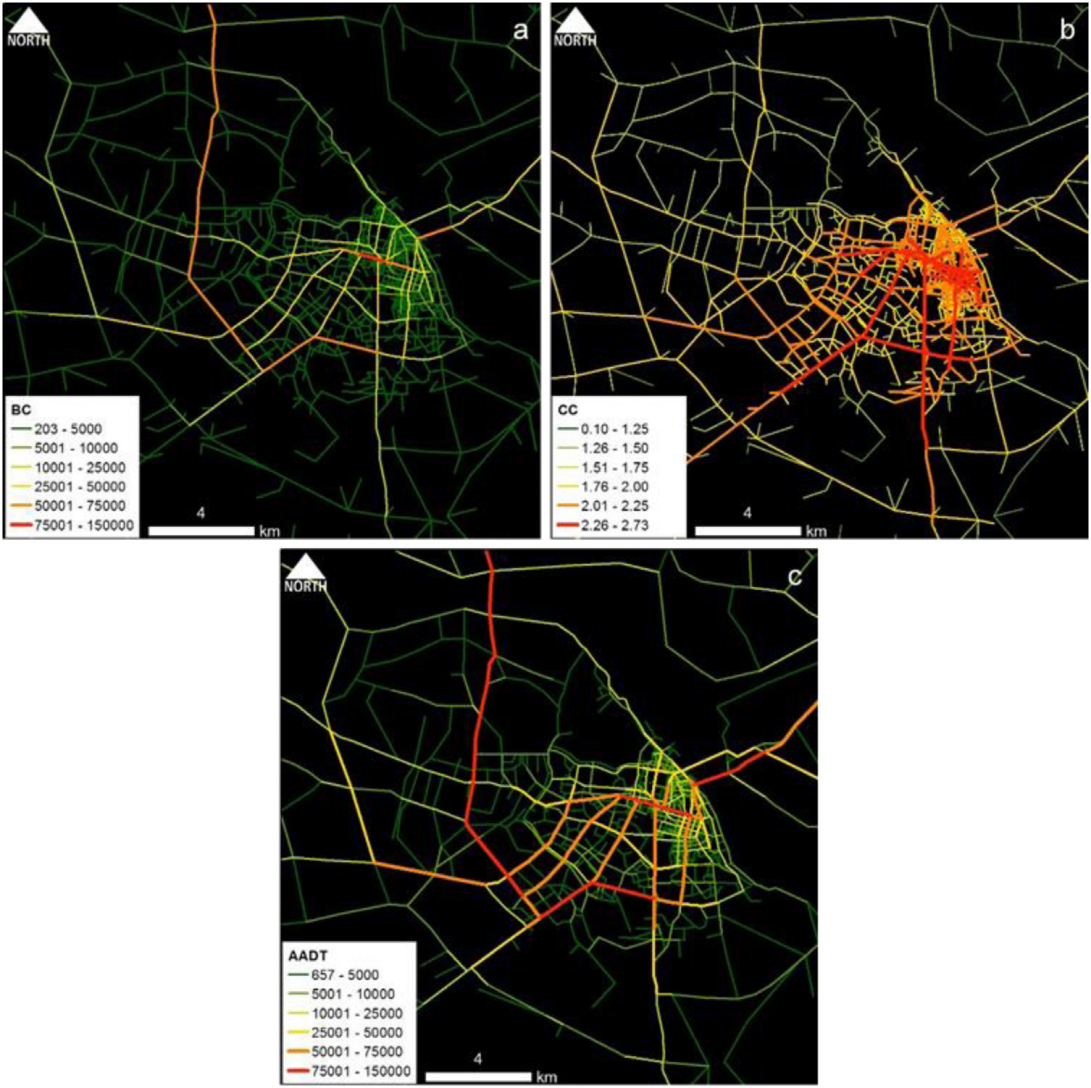
Hanoi case study area: Spatial distribution of (a) BC_(PD)_ and (b) CC_(PD)_ and (c) estimated AADT.

**Fig. 4:**
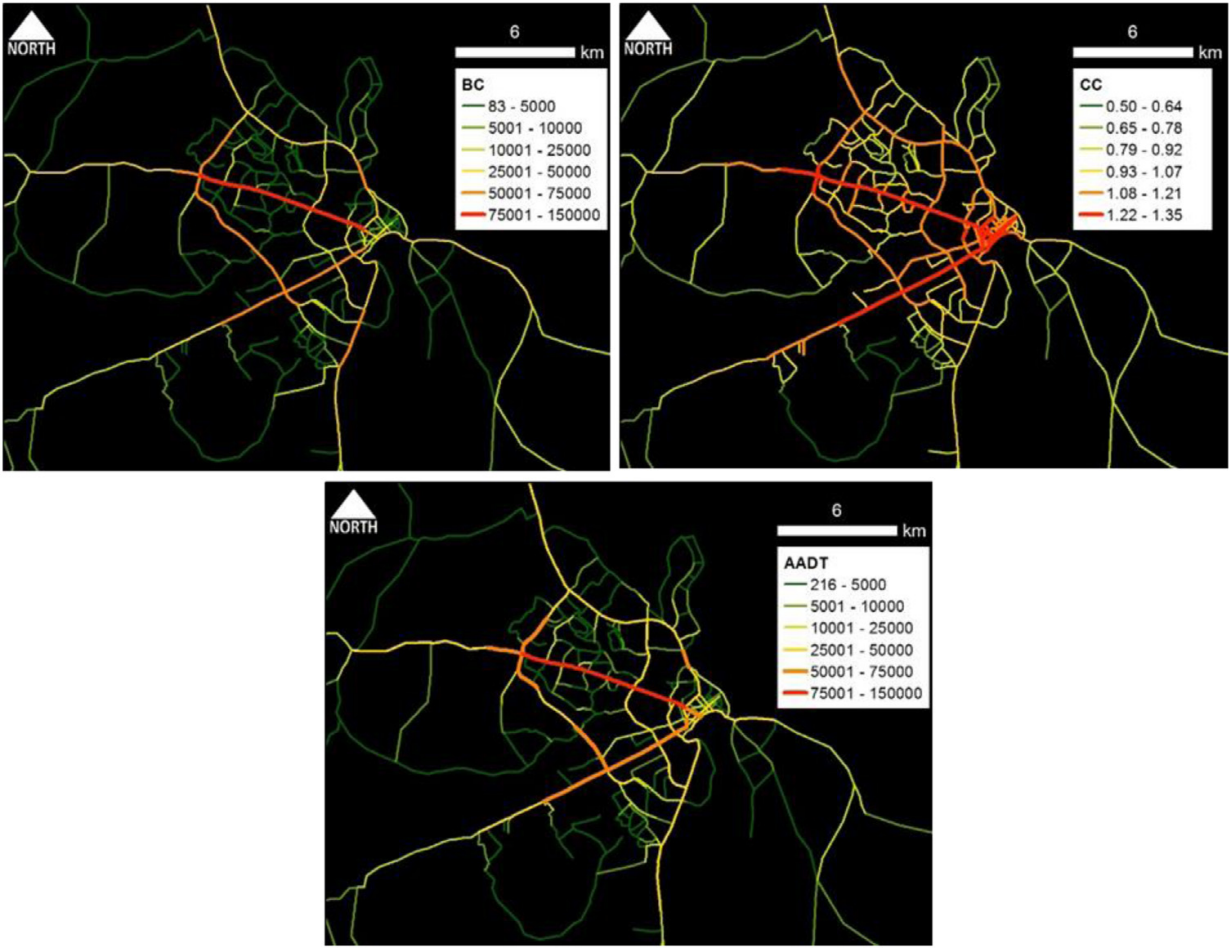
Dares Salaam case study area: Spatial distribution of (a) BC_(PD)_ and (b) CC_(PD)_ and (c) estimated AADT.

**Fig. 5:**
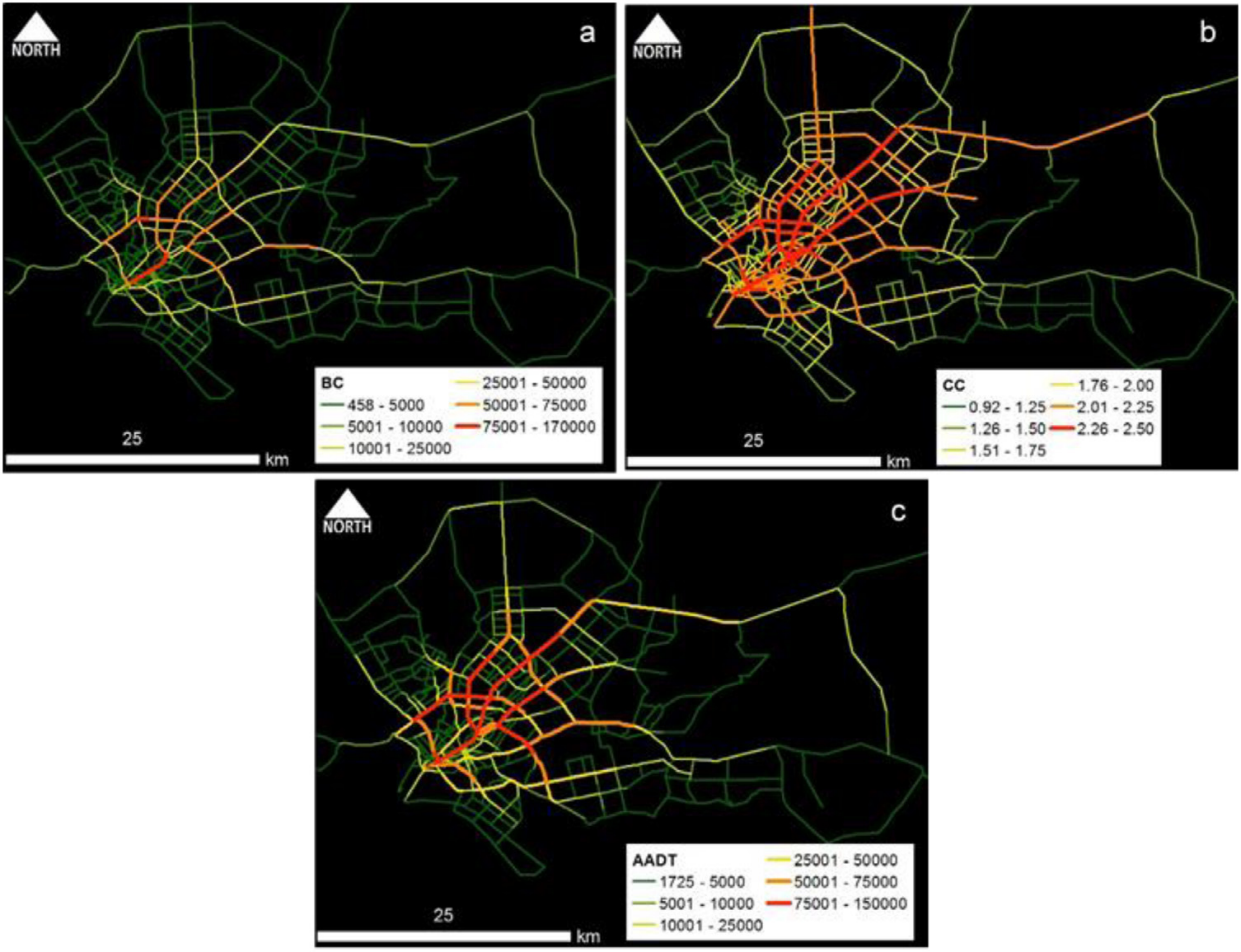
Karachi case study area: Spatial distribution of (a) BC_(PD)_ and (b) CC_(PD)_ and (c) estimated AADT.

One notable fact was that the coefficient values for BC and CC are much similar in all five case study areas. It further strengthens the reliability of the proposed centrality-based method to model traffic volume in any geographical area rather being specific to the tested case studies. Spatial distribution maps of centrality values and AADT estimates from the developed models for five case study areas are shown in Fig. 1, Fig. 2, Fig. 3, Fig. 4, Fig. 5. In those figures, red color indicates high-centrality values and high-traffic volumes, and green color indicates the roads with low centrality values and low traffic volumes. Those maps representing the overall picture of traffic flow rather than traffic volumes of few locations are very useful for traffic and transport engineers, and urban planners.

Conceptually, BC represents the effect of pass-by trips whereas CC represents the effect of O-D trips. Part and Partial correlation statistics were utilized in verifying the practical relevance of predictors; BC and CC. As depicted in the comparative maps, traffic volume flow pattern of BC (Figs. 1a–5 a) is much similar to the overall empirical traffic flow patterns (Figs. 1c–5 c). This indicates that the effect of pass-by trips is dominant over total traffic volume in comparison to the O-D trips. As per the partial correlation values also BC can capture about 60% of the total variability of the predictor variable (i.e., empirical data of AADT values). However, the dominance of BC does not undermine the relevance of CC because CC also solely captures the 32%–35% of the variability of the predictor variable.

The models shown in Table 3 were developed based on actual AADT values obtained from a large number of observation locations (N > 500). However, it is not practical to obtain such traffic volume counts from many observation locations to calibrate the model, particularly within the data scarce situations in developing countries. Accordingly, the study performed a ‘repeated random sub-sampling validation’, [7] to identify the minimum number of observations that required in calibrating the model (refer Table 5). Trained data sets of increasing class sizes were randomly selected for calibrating the model and the accuracy was assessed using the rest of the validation data.

**Table 5:** Minimum number of observations required for calibrations of the model.

Number of Observations for calibration (Training set)	Colombo	Phnom Penh	Hanoi	Karachi	Dares Salaam
	RMSE	RMSE	RMSE	RMSE	RMSE
10	83%	88%	72%	129%	87%
20	54%	46%	50%	91%	34%
30	41%	30%	30%	19%	26%
40	29%	23%	22%	17%	18%
50	23%	22%	20%	15%	18%
60	21%	21%	18%	14%	18%
75	19%	21%	16%	14%	18%
100	19%	18%	16%	14%	18%
500	19%	18%	16%	14%	18%
750	19%	18%	16%	14%	–
1000	19%	18%	16%	14%	–
1500	19%	–	16%	14%	–

**Fig. 6:**
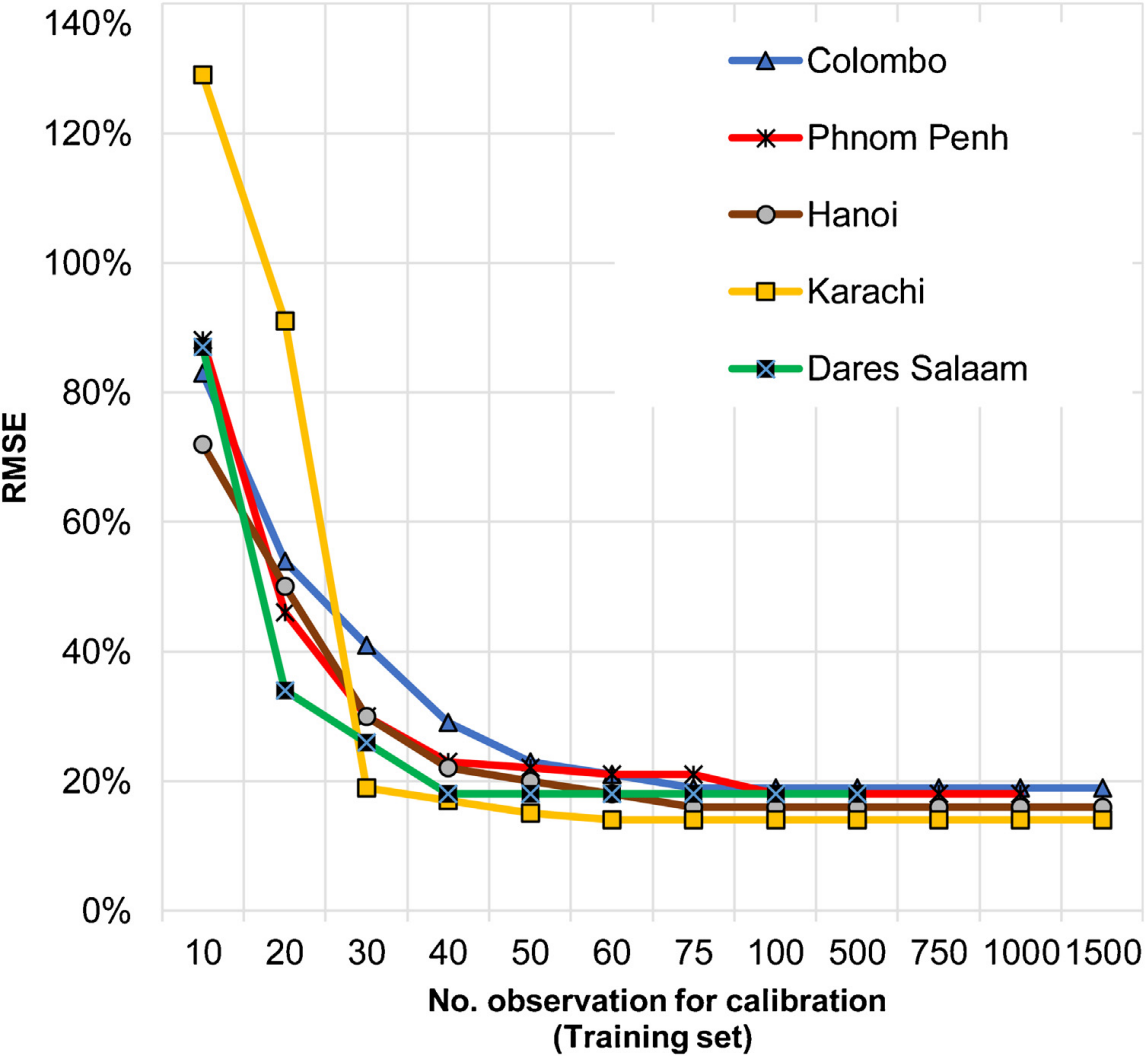
Variation of RMSE values according to the number of observations of the calibration sample.

The results suggest that (refer Table 4), after about 40 observations, RMSE achieves the acceptable level (RMSE < 30%). Fig. 6 graphically illustrates how sensitive the model is to fluctuations in the number of traffic volume observation points (i.e., the size of the training set) on which it is calibrated. As depicted in Fig. 6, Karachi reported an absurd error with the first two subsets of training data but provided an acceptable result after increasing the observations points up to 25. In other case cities also a training set of 25 observations points dramatically reduced the error. The results revealed that the model provides a reasonable accuracy after about 40 observations. This is a far less data requirement compares to the other AADT estimation methods in practice.

Further, this analysis provided an additional check on the uncertainty of the model and how the number of actual observation values may impact on the estimation of AADT values. It indicates that the model could be calibrated by using a little number of observation points resolving time-consuming and expensive data collection constraints.

## Conclusion and recommendation

The key contribution of this study is a developed methodology to model vehicular traffic volume by road segments based on the notion of network centrality. The model composed of two centrality measures which are able to capture both pass-by-trips and O-D trips. Further, the study introduced ‘path distance’, a measure of distance which was derived from the road type and metric distance, to compute centrality values. The combined effect of the hierarchy of the road type and metric distance can well account the mobility characteristics and the roadway characteristics of the road network in measuring the distance. The proposed method does not demand extensive land use, O-D trip data, and traffic count data. It requires only a map of the road network along with a minimal set of actual AADT observations. It also can be implemented by utilizing publicly available network analysis software. Further, the proposed method replaces all four stages of the traditional transport model with accuracy on a par with the international standards. Hence, the proposed method can be considered as a speedy, technically feasible and financially affordable tool to practice. The model is sensitive to the changes of the road network structure and roadway characteristics; and able to be utilized for scenario analysis. The next novel feature is that the proposed method can model vehicular traffic volume in a macro scale road network with a high level of detail up to the road segment level.

The proposed method is tested and validated in four Asian cities and one African city. Five case study areas manifest a unique road pattern which enables to investigate the applicability of the proposed method in diverse geographical areas. The study developed centrality-based models to estimate road segment level AADT with an acceptable level of accuracy (R^2^> 0.90, MAPE < 20% and RMSE < 30%) for all five case study areas. The proposed formula is practice-ready for five case study areas and can easily be employed to estimate AADT of all road segments. Further, the study found that the centrality based AADT estimation model can be calibrated by using a little number of observation points (N < 40) with an acceptable level of accuracy. Hence, the study strongly recommends the proposed network centrality-based traffic volume modeling approach to be applied in modeling traffic volumes in any geographical area. However, when applying this model elsewhere, it is recommended to recalibrate the parameters, without modifying the model structure and the generic method of computing centrality. This study has validated the model utilizing the empirical data on Annual Average Daily Traffic (AADT). Hence, the validation does not explicitly account the seasonal variations of traffic volumes and the daily peaks flow. Further studies are required to test the sensitivity of this model to such fluctuations and congestions propagation. Centrality-based measures in the domain of transport planning still urge for dynamic models. The key constraint in developing dynamic models in developing countries is the lack of real-time traffic flow data for validation. Until such time as the data constraints are overcome and the methods are developed, the proposed method can still be employed in designing hourly volumes based on the peak hour factor as per the highway capacity manual (FHWA). Though there were several attempts on estimating traffic volume based on centrality, most of the practitioners hesitated to employ them due to the inadequacy of accuracy. The proposed method has developed a centrality-based traffic volume estimation model on a par with the international AADT modeling standards. Hence, transport planners and engineers can employ this new method to estimate AADT values in applications such as maintenance, network improvements and traffic management; and model traffic volume in different road network scenarios. Further, this can be utilized as a strategic planning and investment tool for scenario building, and impact analysis of traffic volume and land uses. This method is highly recommended for assignments carry out in developing countries as well as areas where sophisticated multi-step travel demand model is not affordable to implement due to data and cost constraints.
